# Size and specimen-dependent strategy for x-ray micro-ct and tem correlative analysis of nervous system samples

**DOI:** 10.1038/s41598-017-02998-1

**Published:** 2017-06-06

**Authors:** P. Parlanti, V. Cappello, F. Brun, G. Tromba, R. Rigolio, I. Tonazzini, M. Cecchini, V. Piazza, M. Gemmi

**Affiliations:** 1 NEST, Scuola Normale Superiore, Piazza San Silvestro 12, I-56127 Pisa, Italy; 2 Center for Nanotechnology Innovation @NEST, Istituto Italiano di Tecnologia, Piazza San Silvestro 12, I-56127 Pisa, Italy; 3 National Research Council - Institute of Nanotechnology (CNR Nanotech) c/o Dipartimento di Fisica, Università Sapienza, Piazzale Aldo Moro 5, I-00185 Rome, Italy; 4 Dipartimento di Ingegneria e Architettura, Università degli Studi di Trieste, Via A. Valerio 10, I-34127 Trieste, Italy; 5 Elettra - Sincrotrone Trieste S.C.p.A., S.S. 14 km 163.5 in Area Science Park, I-34149 Basovizza, Trieste Italy; 6 Dipartimento di Medicina e Chirurgia, Experimental Neurology Unit, Università degli Studi di Milano-Bicocca, Via Cadore 48, I-20900 Monza, Italy; 7 NEST, Scuola Normale Superiore and Istituto di Nanoscienze - CNR, Piazza San Silvestro 12, I-56127 Pisa, Italy; 8 Fondazione Umberto Veronesi, Piazza Velasca, I-20122 Milano Italy

## Abstract

Correlative approaches are a powerful tool in the investigation of biological samples, but require specific preparation procedures to maintain the strength of the employed methods. Here we report the optimization of the embedding protocol of nervous system samples for a correlative synchrotron X-ray computed microtomography (micro-CT) and transmission electron microscopy (TEM) approach. We demonstrate that it is possible to locate, with the micrometric resolution of micro-CT, specific volumes of interest for a further ultrastructural characterization to be performed with TEM. This approach can be applied to samples of different size and morphology up to several cm. Our optimized method represents an invaluable tool for investigating those pathologies in which microscopic alterations are localized in few confined regions, rather than diffused in entire tissues, organs or systems. We present a proof of concept of our method in a mouse model of Globoid Cells Leukodistrophy.

## Introduction

A disease can be considered multifocal when it affects different organs and tissues, such as multiple sclerosis, with the immune cells infiltrating different regions of the central nervous system (CNS)^1, 2^, and a broad spectrum of cancers that can affect different regions of the same organ (e.g. multifocal and multicentric breast cancer)^3^ or different organs through the metastatic process.

The comprehension of the mechanisms responsible for the onset of these pathologies requires a characterization of macroscopic districts at sub-cellular scale. Nevertheless, often no a priori information is available regarding the localization of the regions of interest for the ultrastructural analysis. In this case, the only possibility would be a systematic characterization of the whole sample at the highest resolution, a challenging and time consuming process. Usually ultrastructural characterization is carried out by transmission electron microscopy (TEM, the standard technique for this purpose) on few tens of nanometres thin sections. Therefore the full characterization of a macroscopic district requires serial sectioning of the whole sample, a procedure which can suffer from serious problems like the loss of a part of the sample during the sectioning process^4^. On the other hand, a global view of a macroscopic district can be also obtained with integrated systems which combine scanning electron microscopy imaging (SEM) with a sectioning system directly embedded in the instrument. Examples are dual-beam instruments in which a focused ion beam (FIB) column is used to erode and polish sequential sections of the sample and a SEM column is used for imaging^5^, or a SEM equipped with 3D view module which allows ultramicrotome sectioning directly on the SEM chamber^6^. All these serial sectioning methods generally require cutting the whole sample, while the global view is obtained through the analysis of the entire collected stack without any previous screening of the regions containing the information of interest. These approaches are then in principle feasible but very costly and cannot become routine techniques.

In this study, we propose a different approach based on the correlative philosophy exploting two different techniques working at different resolutions: the first one furnishes a global view of the sample at low resolution to detect and select specific volumes of interest (VOIs), while the second one returns the ultrastructural characterization of the selected VOIs.

We selected X-ray computed microtomography (micro-CT) as the low-resolution technique that allows obtaining a 3D non-destructive rendering of a macroscopic sample with micrometric resolution, and TEM as the high-resolution technique. Our procedure involves the identification of several micron VOIs in a reconstructed 3D volume and their localization in the sample to cut ultrathin sections for TEM analysis exactly across those VOIs.

First, we set up a specific chemical protocol that could ensure a good contrast in micro-CT and a high ultrastructural preservation for TEM observations, and that can could be tuned to fix a wide range of sample sizes, from mm^3^ to cm^3^ volumes^7^.

Examples of a similar procedure has been reported for both inorganic materials^8^ and tissue specimens^9, 10^. However, there is no discussion on the optimization of a size and morphology dependent embedding method for correlative analysis. Our work presents, for the first time, a systematic comparison of sample preparation procedures to optimize protocol strategies according to the type and the size of the analyzed tissue.

The validation of the different chemical protocols and the entire correlative procedure has been first tested on the sciatic nerves of Twitcher homozygous mice (TWI), a well-characterized model of Globoid Cell Leukodistrophy (GLD, also known as Krabbe disease). Krabbe disease is a neurodegenerative disease characterized by infiltration of immune system cells (globoid cells) into the nervous system driven by the inflammatory process^11, 12^. This pathology represents a perfect proof of concept sample, since those globoid cells are the main hallmark of the disease^13^ and they randomly infiltrate the target organs.

The correlation technique was further validated on bigger and more complex samples (mice and rat spinal cords) prepared using our specific protocols.

## Results

### Protocol optimization

**Figure 1:**
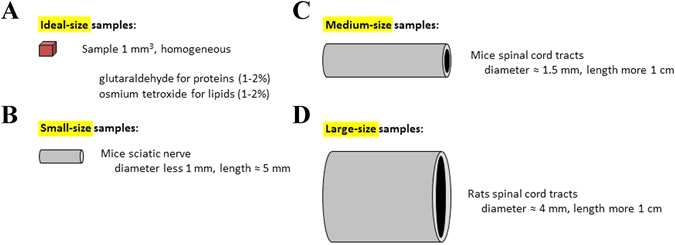
Sample sizes classification. (**A**) Ideal-size sample - cellular pellet; (**B**) small-size sample - mice sciatic nerves; (**C**) medium-size sample - mice spinal cord tracts; (**D**) large-size sample - rat spinal cord tracts. The sizes reported in the figure are proportional to the real dimensions of the samples.

The ideal size of a biological sample for the TEM analysis is around 1 mm^3^ in volume (Fig. 1A). Our goal has been to settle on protocols for preparing larger samples up to 1 cm^3^ in volume (Fig. 1B–D) and to make the embedding protocol for TEM analysis compatible with micro-CT observations.

Because the generation of a 3D map by means of micro-CT is the pivotal step for the individuation of specific VOIs within the sample, the first evaluation of our protocols has been performed on X-ray tomography images and 3D renderings.

#### Protocol optimization for micro-CT

**Figure 2:**
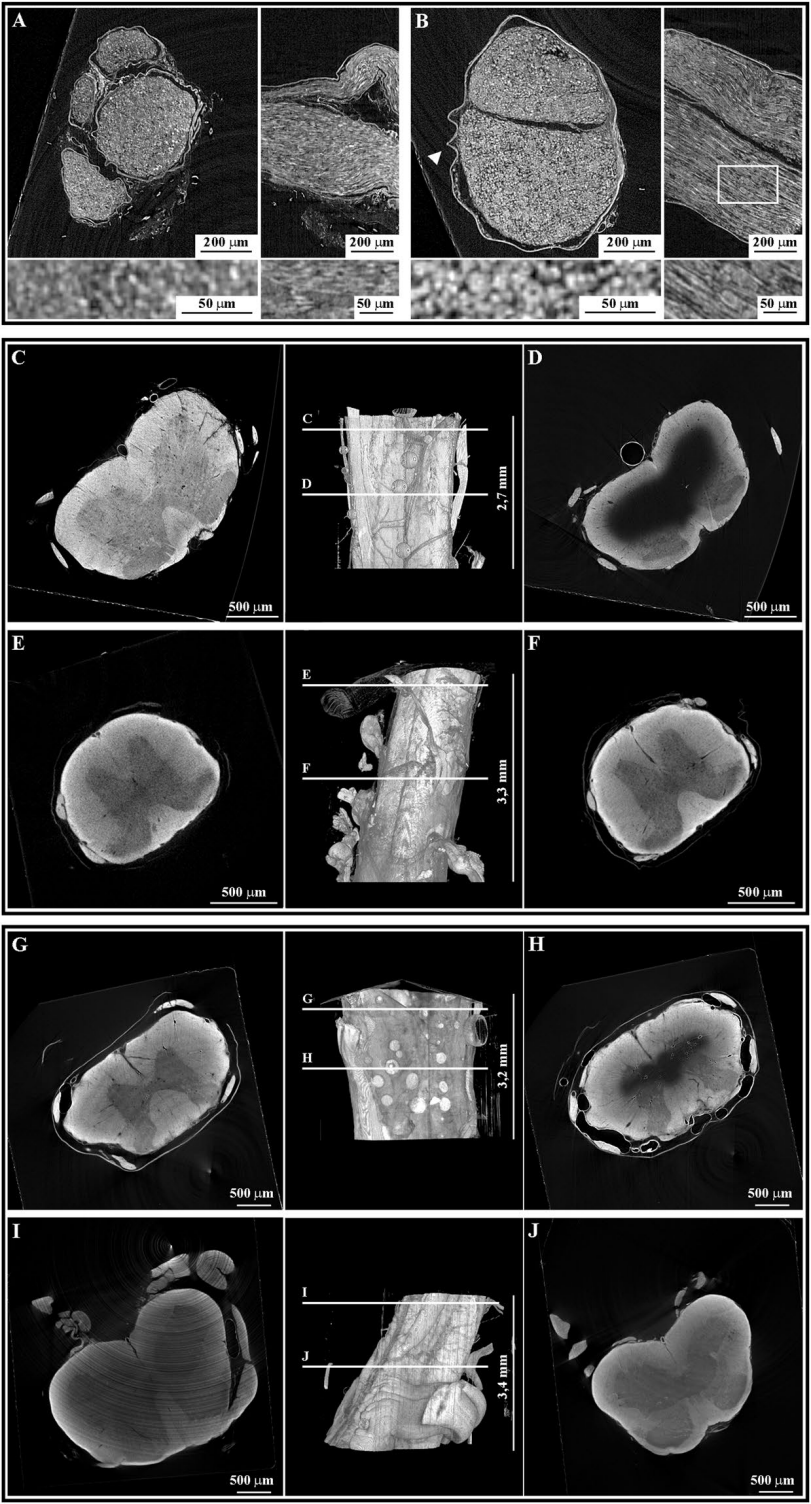
The protocol optimization: micro-CT results. Upper panel: sciatic nerves prepared with (**A**) the ideal-size or (**B**) the optimized small-size protocol. Middle panel: mice spinal cord tracts prepared with (**C**,**D**) the small-size or (**E**,**F**) the optimized medium-size protocol. Lower panel: rat spinal cord tracts prepared with (**G**,**H**) the medium-size or (**I**,**J**) the optimized large-size protocol. For sciatic nerves are reported representative xy and yz projections and their higher magnification in the bottom lines. For mice and rats spinal cord tracts are shown a representative virtual slice of the top part of the sample, a 3D rendering of the whole sample and a deeper virtual slice.

Depending on their size, we have distinguished 4 groups of samples (Fig. 1 and Supplementary Figure 1): (1) an ideal-size sample, which is 1 mm^3^ in volume and morphologically homogeneous (Fig. 1A); (2) a small-size sample (Fig. 1B - the mice sciatic nerve), which has a comparable planar surface but it is longer; (3) a medium-size sample (Fig. 1C - cervical/thoracic/lumbar tract of the mice spinal cord), which is larger in the planar surface and longer, with two concentric and morphologically not homogeneous regions; (4) a large-size sample (Fig. 1D - cervical/thoracic/lumbar tract of the rat spinal cord), morphologically similar to the medium-size sample, but twice larger in volume.

As described in the following, we tested several combinations of fixative and embedding conditions to define the best “sample and size-dependent” protocol to be used for our correlative purpose. Starting from common procedures^14^ usually described for the plastic embedding of nervous system (“Ideal-size protocol” column in Table 1), we identified few steps - protein fixation, lipid fixation and heavy metals staining - that could be critical, and must be optimized, for our purposes.

**Table 1:** The specific embedding procedure for each sample size.

	Ideal-Size Protocol (ideal sample)	Small-Size Protocol (mice sciatic nerves)	Medium-Size Protocol (mice spinal cords)	Large-Size Protocol (rats spinal cords)
**Protein Fixation (perfusion)**	Para 4% + Gluta 0.1% in PBS	Para 4% + Gluta 0.1% in PBS	Para 4% + Gluta 0.1% in PBS	Para 4% + Gluta 0.5% in PBS
**Protein Fixation**	Overnight Gluta 2% in NaCaco Buffer	Overnight Gluta 2% + Para 1% + Form 1% in NaCaco Buffer	Overnight Gluta 2.5% + Para 1% + Form 1% in NaCaco Buffer	Overnight Gluta 2.5% + Para 1% + Form 1% in NaCaco Buffer
**Lipid Fixation**	1 h OsO_4_ 1% + K_3_Fe(CN)_6_ 1% in NaCaco Buffer	1 h OsO_4_ 1% + K_3_Fe(CN)_6_ 1% in NaCaco Buffer	8 h OsO_4_ 1% + K_3_Fe(CN)_6_ 1% in NaCaco Buffer	8 h OsO_4_ 2% + K_3_Fe(CN)_6_ 1% in NaCaco Buffer
			Overnight OsO_4_ 1% in NaCaco Buffer	Overnight OsO_4_ 1% + K_3_Fe(CN)_6_ 1% in NaCaco Buffer
**Contrast**	1 h UA 3% in ethanol 20%	1 h UA 3% in ethanol 20%	1 h UA 3% in ethanol 20%	1 h UA 3% in ethanol 20%

The table reports all the embedding size-dependent procedures we have specifically modified for the best result of our correlative analysis.

Abbreviations: Gluta = Glutaraldehyde/Para = Paraformaldehyde/Form = Formaldehyde/NaCaco Buffer = Sodium Cacodylate Buffer 0.1 M pH 7.4/UA = Uranyl Acetate/PBS (Gibco, Life Technologies) pH 7.4.

#### Small-size samples (mouse sciatic nerves)

For the embedding of the small-size samples we tested different aldehydes mixtures in the protein fixation steps (“Protein fixation”, first and second row in Supplementary Table 1), to improve the infiltration of the reagents inside the sample^12, 14–16^. We added potassium ferricyanide to osmium tetroxide (OT) in the lipid fixation step to enhance its oxidant power^16^ (“Lipid fixation”, Supplementary Table 1, row 3).

From the comparison of micro-CT collected on the tested protocols (Supplementary Table 1) we found that the sciatic nerve treated with the “Small-size protocol” (see Table 1) had the best X-rays contrast revealing fine details of its internal structure: several small features such as infiltrating globoid cells, small capillaries lumen and axons (Fig. 2B) were clearly visible. Conversely, those fine details could be hardly identified in the other tested protocols (see the comparison with two virtual projections of the sample prepared with the ideal-size protocol in Fig. 2A).

The key point for obtaining the best protocol was the addition of paraformaldehyde and formaldehyde to the glutaraldehyde solution in the second step of protein fixation (Table 1 and Supplementary Table 1). This modification improves fixation in the inner part and the preservation of tissue morphology. Unexpectedly, we found better results in the micro-CT quality when the samples were prepared with glutaraldehyde 2% + paraformaldehyde 1% + formaldehyde 1%, compared with glutaraldehyde 2% + paraformaldehyde 2% or glutaraldehyde 2% alone. A detailed investigation of this peculiar behavior is beyond the scope of this work. At the same time, the use of the OT with the potassium ferricyanide lead to better membrane preservation and better maintenance of myelin sheaths, as previously described by Langford and Coggeshall^17^.

#### Medium-size samples (mice spinal cord tracts)

Next, we moved to test the optimized protocol for the sciatic nerve on a whole tract of the mice spinal cord. We observed that this protocol did not provide a good contrast of the inner part of the sample (Fig. 2C compared to D). In fact, starting from a depth of about 500 µm from the top, only the outer part of the spinal cord was well stained. We explain this result as a consequence of the inability of the solutions to diffuse in the inner part of such big samples.

Moreover, while the nerve can be considered as a tube made of myelinated axons, the spinal cord has a more complex structure which is ideally formed by two concentric layers, with the white matter mainly placed peripherally to the grey matter represented the inner part of the spinal cord (respectively the grey and the black part in Fig. 1C,D). Therefore, the reagents used during the different embedding procedure steps of embedding procedure could react differently with these two morphologically distinct parts of the tissue.

Thus, we modified the fixative and contrast protocols (see supplementary Table 2) by increasing the time of the OT step and testing different OT, reduced-OT and uranyl acetate mixtures. Indeed, a longer OT treatment could have been critical in overcoming the slow diffusion of the solutions.

The move was successful: we obtained an optimal micro-CT contrast and a homogeneous staining of the coronal sections for the whole depth analyzed (more than 1 cm length; see Fig. 2E,F that refers to a 3.3 mm tract of the sample) with a double step of longer treatment using reduced-OT and OT respectively (“Medium-size protocol” in Table 1). We can therefore speculate that the efficiency of the small-size protocol was limited by the diffusion factor with the solutions not reaching the inner part of the tissue.

#### Large-size samples (rat spinal cord tracts)

The medium-size protocol was used as the basis to prepare the larger rats spinal cord samples. Similarly to what we observed for the medium-size sample prepared with the small-size protocol, the medium-size protocol did not ensure a homogeneous contrast in the sample: while the top portion of the sample was correctly stained (Fig. 2G), the inner part of the tissue appears “empty” in its central portion (Fig. 2H).

By following the strategy previously described, we directed our efforts in changing the time and the concentration of the reagents used in the “Lipid fixation” step (supplementary Table 3). This strategy was effective, leading to a decrease in the size of the missing contrast region, in particular when the protocol involved longer treatment with OT or a higher concentration of reduced-OT.

A uniform contrast between the inner and outer part of the spinal cord (Fig. 2I,J) for the whole analyzed depth (more than 1 cm length) was obtained in a two-step treatment with variable concentration of reduced-OT, higher in the first step and lower in the second one (“Large-size protocol” in Table 1).

#### Evaluation of the ultrastructural preservation

The optimized protocols for micro-CT allow identifying specific VOIs within the sample, therefore solving the first issue of our correlative method. However, a well-defined micro-CT contrast does not necessarly assure an adequate preservation of the tissue structure at the nanometric scale, the level at which the selected VOIs would give us access to sub-cellular mechanisms of the pathology. Therefore, the second step for the validation of our protocols for micro-CT involved the evaluation of their efficiency in preserving the ultrastructure of the tissues as analyzed by means of TEM imaging.

#### Small-size samples (mice sciatic nerves)

**Figure 3:**
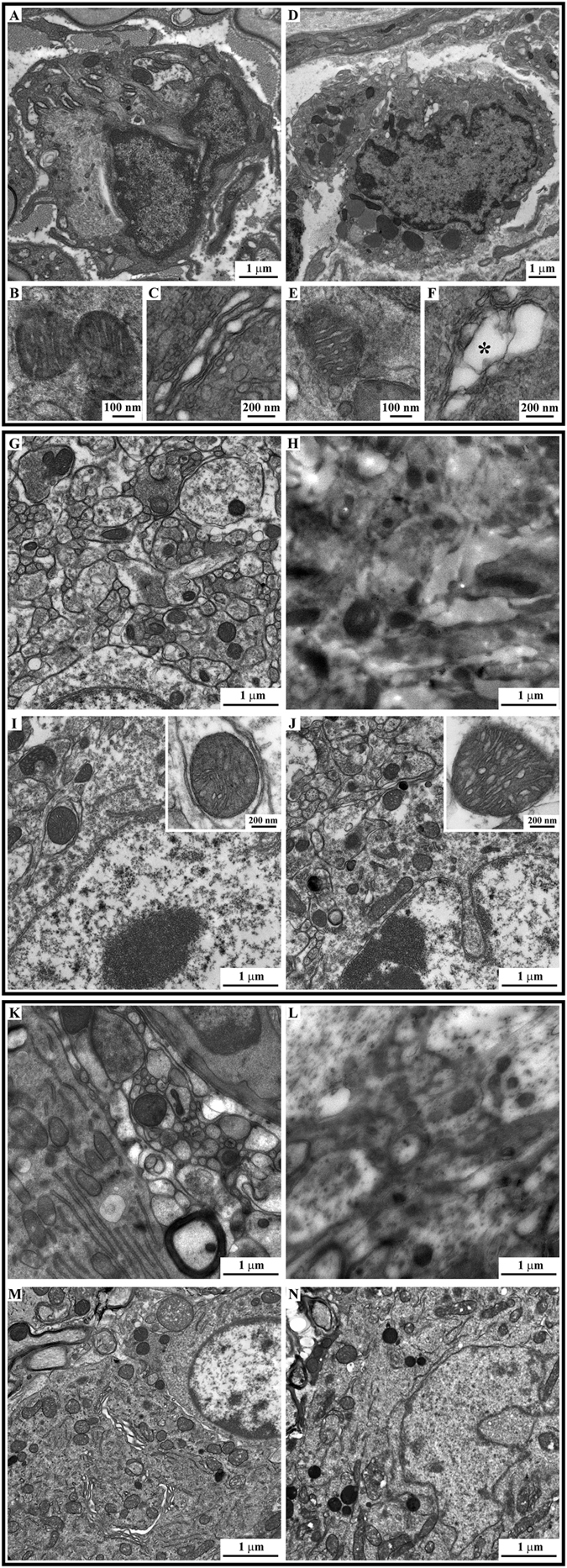
The protocol optimization: ultrastructural results. Upper panel: respectively, electron micrographs of a globoid cell (pathological hallmark), of a mitochondria and of a Golgi apparatus derived from a sciatic nerve treated with the ideal-size protocol (**A–C**) or with the optimized one (**D–F**). The ultrastructure is well preserved with both chemical procedures even if a mild swelling has been observed only few time in samples prepared with the optimize one (*). Middle panel: Electron micrographs of the ventral horns of two mice spinal cords prepared with the small-size protocol (**G**,**H**) and with the optimized protocol for medium-size samples (**I**,**J**). Representative images of the (**G** and **I**) outer portion and of the (**H** and **J**) central part of the samples −500 µm beyond. Ultrastructural alterations, clearely visibile in the sample prepared with the small-size protocol (**H**), are completely abolished using the optimized protocol (**I** and **J** and insets). Lower panel: Electron micrographs of the ventral horns of two mice spinal cords prepared with the small-size protocol (**K**,**L**) and with the optimized protocol for medium-size samples (**M**,**N**). Representative images of the (**K** and **M**) outer portion and of the (**L** and **N**) central part of the samples −500 µm beyond. The ultrastructure of samples prepared with the optimized protocol is extremely well preserved at every depth analyzed, while this is completely loss in the sample prepared with the medium-size protocol (**L**).

As a reference we used a mice sciatic nerve prepared with the ideal-size protocol where the ultrastructure preservation is optimal. We found that our samples prepared with the small-size protocol shows a very good ultrastructure which allows the visualization of extremely precise details such as cytosolic organelles and their lipid membranes (Fig. 3D–F), with a quality comparable to the samples prepared with the ideal-size protocol (Fig. 3A–C). Only slight alterations of the membranes of subcellular districts were reported (Fig. 3F). All together the results indicate that the small-size protocol is the best compromise between the high quality of the micro-CT analysis and the good ultrastructural preservation that allows characterization of the sample at sub-cellular level.

#### Medium-size samples (mice spinal cord tracts)

As described before, when the protocol was not optimized based on the sample-size, the solutions for the embedding procedure could not diffuse in its inner part beyond 500 µm in depth from the outer surface. For this reason, we evaluated ultrastructure of the samples at this level for the non- and optimized protocols (respectively, small-size protocol in Fig. 3G,H and medium-size protocol in Fig. 3I,J and insets).

Our optimized protocol (medium-size protocol) effectively preserved ultrastructure in the central region at each depth in the sample (top and higher depth, Fig. 3I and J respectively). Conversely, the non-optimized approach (small-size protocol) was efficient only in the first hundreds of microns of the sample, while beyond the tissue architecture was completely lost (Fig. 3G vs. H).

#### Large-size samples (rat spinal cord tracts)

We clearly highlighted that a protocol optimized for smaller samples can not be applied to larger ones while maintaining the same effectiveness in the ultrastructural preservation. For mice spinal cords, we observed that the protocol optimized for the medium-size samples was not suitable for the evaluation of the sample morphology at a depth greater than 500 μm (Fig. 3K compared to Fig. 3L). Our optimized protocol for large-size samples, instead, leads to perfect ultrastructure preservation in the first micrometers of the sample (Fig. 3M) and in its central portion, beyond 1 mm in depth (Fig. 3N).

### Correlation procedure

The next step was the setup of a correlation procedure that was tested on sciatic nerves prepared with the optimized small-size protocol.

**Figure 4:**
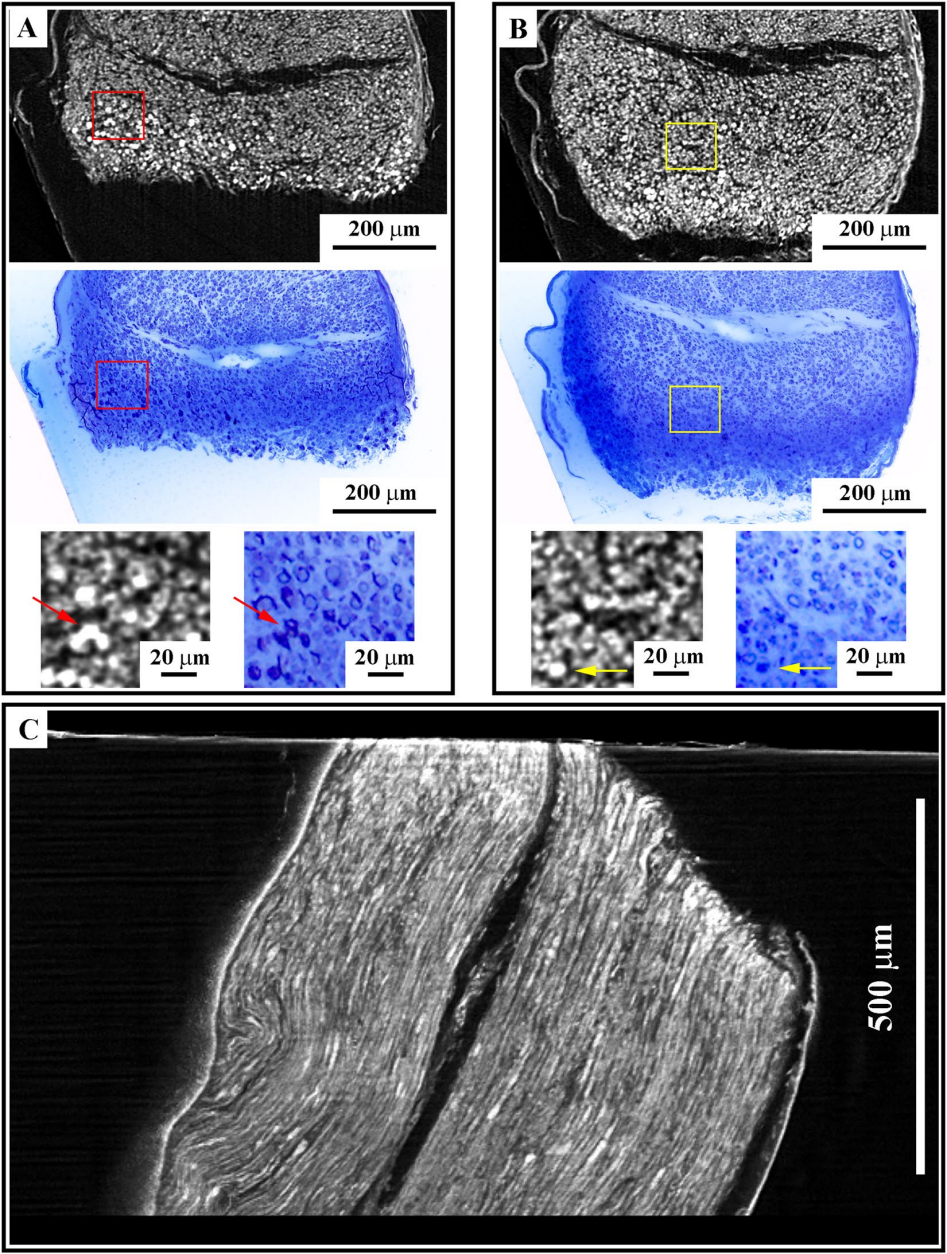
The correlation procedure. (**A**) A representative virtual slice (first row) correlates perfectly with the corresponding optical image (second row). In the bottom row is reported the enlargements of the regions boxed in each virtual and optical section. Arrows indicate some of the correlation points used for the alignments of virtual and optical sections. (**B**) The alignment is preserved at different depths. (**C**) A yz view of the sample in micro-CT.

Therefore, we cut arrays of sections normal to the rotation axis of the micro-CT scan (Fig. 4), to check: (1) if the details observed in micro-CT virtual slices could be correlated with those detected in optical and TEM images of real sections and (2) if this correlation could be traced over a certain thickness.

The nerves were sectioned in arrays of serial sections having a thickness of 500 nm each, for a total thickness of 10 µm, followed by few thin sections cut for TEM analysis (90 nm thickness per section, about 1 µm of whole volume). The procedure was repeated at different depth of the same nerve (Fig. 4C). Serial sections were observed using an optical microscope and the images were collected for the correlation with virtual slices (Fig. 4A,B).

The first step of the correlation procedure consisted in the alignment of the virtual section normal with the cutting direction (Fig. 4A,B). The tomographic rotation axis was selected to be the as close as possible to the main axis of the samples (i.e. orthogonal to the sectioning direction), thus we sought a correspondence between the first optical image of the first array and virtual sections, whose normal was few degrees away from the tomographic axis. Once a good agreement was found, the alignment was furthered refined by advancing 10 µm in the virtual sectioning to the last optical image. After the alignment of both these sections was carried out, all the optical images of intermediate sections could be retrieved just by displacing the virtual sectioning accordingly to the computed distance, taken for granted that the minimum voxel size was 2.15 µm.

The first ultrathin section located at the end of the array volume was observed in the TEM at low magnification (250X) by collecting images of the whole section in order to generate a 2D map comparable with an entire virtual slice. The obtained map perfectly correlated with the last virtual slice of the array. The same section was also observed at higher resolution to check the quality of the sample ultrastructure. We could conclude that after a preliminary alignment of the cutting direction within the virtual volume, any optical or TEM image of the whole section array could be correlated with a corresponding virtual slice in the stack: this constituted the first step toward a correlative method.

We also validated this alignment procedure on samples of greater size looking for precise details in mice and rat spinal cords (Supplementary Figure 2 first and second panel, respectively).

As shown in the figure, it was possible to define the same VOIs in micro-CT (Supplementary Figure 2A) and in real sections (Supplementary Figure 2B) in medium-size samples: here we identified a confined region of blood vessel branching in micro-CT images (Supplementary Figure 2C,E), which was further characterized in optical images (Supplementary Figure 2D,E) and with the TEM (Supplementary Figure 2F,G). This evaluation could represent a useful tool in the study of those pathologies where an alteration of the blood brain barrier causes a cellular extravasation (i.e. multiple sclerosis).

As described for the mice spinal cords, the correlation technique could be applied also to the rat spinal cords prepared with the optimized protocol (large-size protocol). In this case, we aligned the virtual slices (Supplementary Figure 2H,J,L) to the real sections (Supplementary Figure 2I,K,L), and identified the same features, as cells (Supplementary Figure 2N,O), observed at lower resolution in thin sections (Supplementary Figure 2M).

### The correlation technique and pathological hallmarks

As final step, we validated the correlative procedure in detecting special VOIs in a model for GLD (Krabbe disease), which is characterized by globoid cells infiltrating and uniformly distributed within the sciatic nerve (Supplementary Figure 3). Those cells are usually well separated from axons and this feature allowed discriminating them from Schwann cells (SCs) that are always associated with axons. We could easily visualized globoid cells as small-intercalated island between the axons, both in the planar (Supplementary Figure 3A,C) and in the orthogonal (Supplementary Figure 3B,D) views of micro-CT virtual sections.

**Figure 5:**
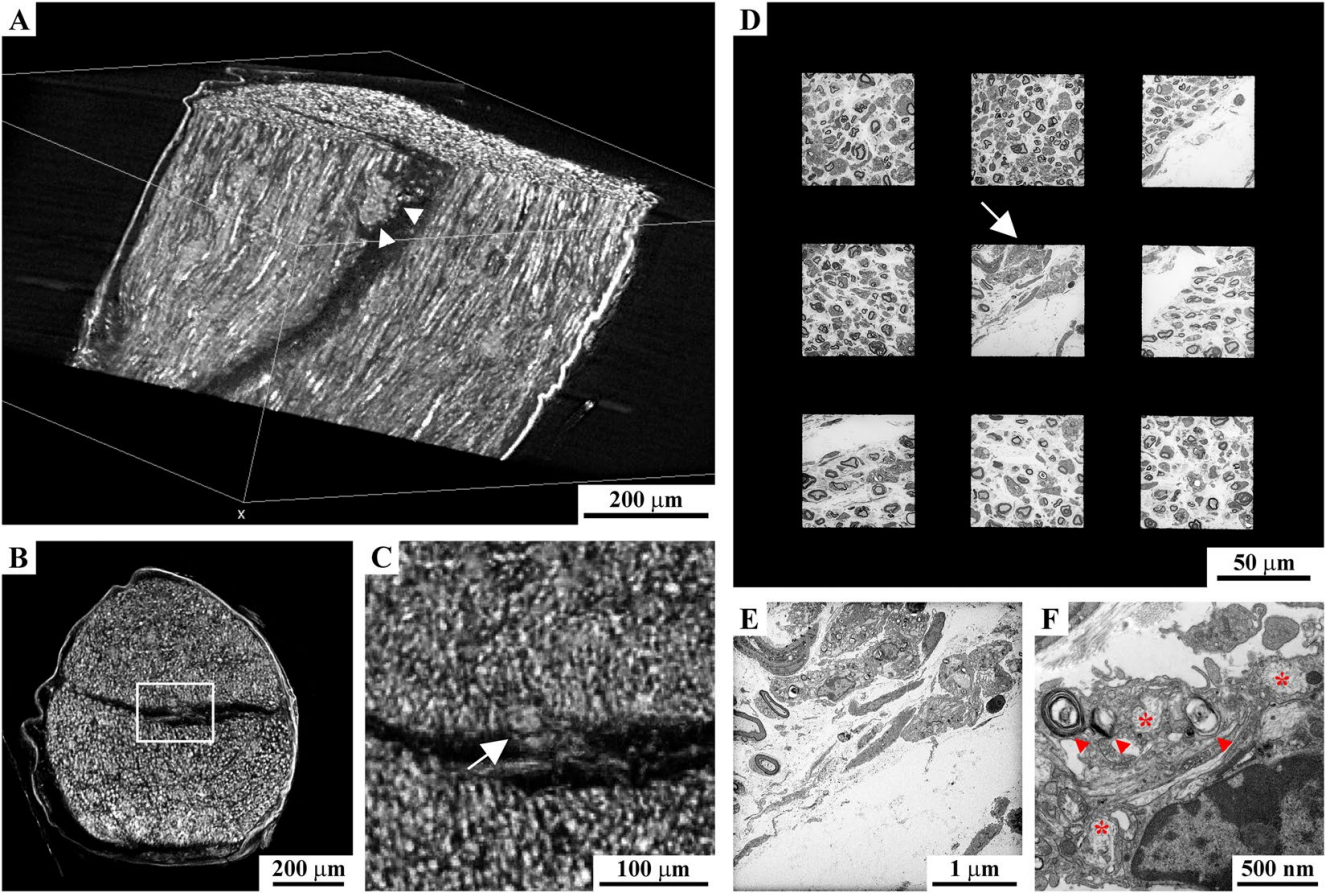
Identification of the same globoid cell in the sciatic nerve in 3D rendering, in virtual slices and in sections for TEM. (**A**) 3D rendering of a portion of sciatic nerve treated with the small-size protocol, in which it is possible to identify an infiltrating cell (white arrowheads). (**B**) Virtual slice of the same sample. (**C**) Higher magnification the boxed region, in which the selected cell is pointed out (arrow). (**D**) Identification of the same cell in 2D TEM map (arrow). (**E**,**F**) Ultrastructural characterization of the selected cell. Arrowheads and * highlight specific morphological characteristics of globoid cells.

After preparing an array of sections for retrieving the cutting direction and the position of the first section in the 3D computed tomography, we identified a single infiltrating cell in the 3D rendering and in virtual slices (respectively, arrowheads in Fig. 5A and arrow in Fig. 5C). By measuring the distance between the last cut section of the alignment array and the selected globoid cell, we could calculate the volume of sample that must be removed before the cutting surface approaches the VOI. We removed 500 nm-thick slices at time at relatively fast speed (5 mm/sec) stopping 10 µm before reaching the VOI, to maintain a safety volume on which checking and finely tuning the registration. After milling, we cut one section to confirm the correct alignment and the position. Then we further proceeded to cut 500 nm until the selected cell was visible in the section at the optical microscope. Finally we prepared an array of thin sections (90 nm) of the whole volume of the cell, and observed at TEM for the ultrastructural characterization (Fig. 5D–F).

Globoid cells, as previous described in Suzuki and Suzuki in 1983, show the morphology of activated macrophages^13^. The main features of these often multinucleated cells is the presence of lysosomes that separately stock cytoskeletal elements or myelin sheaths (respectively, * and arrowheads in Fig. 5F). Several cells can be often observed in close proximity of a blood capillary in those regions that are strongly affected to the pathology, where the degeneration of myelin sheaths is particularly important^12^.

## Discussion

Neurodegenerative disorders are a complex disease family in which a tool for an appropriate analysis could be the crucial access point to the comprehension of pathological process and for the development of new therapies.

Here we propose an innovative tunable method for the correlation of synchrotron X-ray computed microtomography (micro-CT) and transmission electron microscopy (TEM) in the study of neurodegeneration.

Differently from other 3D methods applied to biological and inorganic samples analysis, we combined the non-distructive micro-CT analysis, as VOIs screening procedure, and TEM on specific small volumes. In this way we obtained a virtual map that removes the need for whole sample serial sectioning^4^, or the use of integrated systems such as the FIB-SEM^5^ or the serial block-face SEM^6^, ultimately leading to a great reduction of the experimental time.

The idea of correlative techniques involving TEM and micro-CT imaging has already been reported in the literature^9, 10, 18^. Recently, Handschuch has analyzed a small juvenile bivalve mollusk with micro-CT to obtain a 3D rendering of the sample, which has not been utilized as a map for the specific cutting process. In fact, the sample was entirely cut in thick sections (1.5 μm) for the optical screening which has led them to the identification of the VOI with a further very time-consuming re-sectioned for the TEM analysis^18^.

Moreover, Sengle in 2013 has applied a correlation micro-CT/TEM on sample arising from the mice forelimbs to evaluate the amount of the adipose tissue^10^. However, in this interesting paper, the authors needed to image adipose tissue in the periphery of a mouse forelimb which could be considered a more accessible and less complex sample compared with the nervous system. The authors did not exceeded the total height of 3.2 mm during the micro-CT acquisition, thus their method did not require a fine chemical tuning for the embedding procedure.

Finally, Morales and coworkers presented a correlative method which is very similar to ours, but it has been applied on a small-sample (comparable with our ideal-size one) that arising from the scraping of human nasal mucous^9^. For the above mentioned work, the substantial difference with our procedure depends on morphology, size and accessibility during the sampling. Moreover, the small-size sample they work with allows them to use a single chemical protocol without the need to tune it. The advantage of our approach relies on the possibility to optimize the chemical procedure leading to the possible application of our method to the overall analysis of variable size biological samples.

We have shown that the sample embedding procedure, for the correlative method, is very challenging. The chemical protocol has to be tuned depending on the size and morphology of every specific sample maintaining the ultrastructural preservation (for TEM) and the contrast (for micro-CT).

We have shown that the chemical procedure optimization involves three key steps: (1) protein fixation, (2) lipid fixation, (3) heavy metals staining; finally, we obtained three different optimized protocols (Table 1) that can be exploited for micro-CT and TEM on the nervous system samples and pathologies.

The very detailed 3D reconstruction obtained with micro-CT (Fig. 2) is a guide for the internal structure of a biological district and it could be routinely used as a map to drive the cutting procedure at specific VOIs level for the deeper investigation of the sample. A concrete application has been reported using the Twitcher sciatic nerves for which micro-CT gives a 3D view of the axons arrangements that can be followed through several millimeters for localizing the infiltrating globoid cell (Supplementary Figure 3), whose thin VOIs has been directly cut to investigate tissue and cell morphology at a sub-cellular resolution (Fig. 3 upper panel).

We demonstrated that it may be possible to select, identify and further characterize similar regions within a larger samples such as mice and rat spinal cords.

While in previous works the micro-CT studies were carried out on conventional benchtop systems and scans were taken in absorption imaging modality^10, 18^, in this study the use of synchrotron radiation made it possible to apply phase contrast technique and phase retrieval algortithm to increase image contrast^19^. However, it is reasonable to state that similar results can be obtained with conventional microfocus X-ray sources, although acquisitions are more time-consuming and artifacts may occur in the 3D reconstructions. Therefore the proposed chemical protocols can be used for the correlative method also with easy-to-access laboratory devices.

In conclusion, our work reached two main targets: the development of an optimized embedding procedure for the micro-CT/TEM correlation and the clear potential application of this method to the study of neurodegenerative pathologies. However, this procedure can be applied to the investigation of those pathologies in which microscopic alterations are localized in few confined regions, rather than diffuse in entire tissues and organs. A potential application field is represented by the imaging of regions affected by the metastatic process using micro-CT followed by the cell type identification through ultrastructural analysis with TEM. Moreover, this technique could have clinical application for screening rare pathological events inside bioptical tissues.

## Methods

### Animals

#### WT and TWI mice

Twitcher heterozygous (B6.CE-Galc^twi^/J, Jackson Labs) and wild-type (WT, C57Bl/6J) mice were generously donated by Dr. A. Biffi (San Raffaele Telethon Institute for Gene Therapy, Milan, Italy). Animals were maintained under standard housing conditions and used according to the protocols and ethical guidelines approved by the Ministry of Health (DLGS 26/2014), as per Italian law (Permit Number: 0004419). Twitcher heterozygous mice were used as breeder pairs to generate homozygous Twitcher mice (TWI).

Genomic DNA was extracted from the clipped tails of mice by Proteinase K lysis buffer^20^ as previously described^21^. The genetic status of each mouse was determined from the genome analysis of the twitcher mutation, as reported in Sakai and coworkers^22^. Four TWI male mice at P30 and five WT male littermates were used for experiments, while the heterozygous littermates were retained for the colony maintenance. Surgical procedures for fixation were performed under urethane anesthesia (Sigma, 0.8 ml/hg), and all efforts were made to minimize mice suffering.

#### Wistar rats

Female Wistar rats were purchased from Envigo (175–200 g, San Pietro Natisone, Udine) and housed three per cage, in a limited access animal facility with controlled room temperature and relative humidity, artificial light/dark 12 hour cycle and food/water access ad libitum according to ethical guidelines approved by the Ministry of Health. The animals were deeply anestetized by ketamine/xylazine solution (90–120 mg/kg; 5–10 mg/kg) before surgical procedures for fixation were performed and all efforts were made to minimize rat suffering.

### Chemical preparation

#### Small-size samples

Four P30 (±2) Twitcher and one WT littermate deeply anesthetized mice were sacrificed by perfusion with two different fixative solutions (one TWI with the higher amount of glutaraldehyde (0.5%) in the solution, while all the others with the lower concentration of glutaraldehyde (0.1%)); then, sciatic nerves were extracted and post-fixed for 4 hours in the same fixative solutions.

The TWI sciatic nerves were divided into smaller parts, each one of 3 to 5 mm, and then the samples were treated with 4 different chemical protocols for epoxy resin while the WT one was entirely embedded. The optimized protocol is summarized in the Table 1. The other tested protocols differ with the optimized one in the aldehydes concentrations (Supplementary Table 1).

#### Medium-size samples

Four P30 (±2) Twitcher and five WT littermates (four TWI and one WT were the same animals used for the small-size sample experiments) deeply anesthetized mice, were sacrificed by perfusion with two different fixative solutions; then the entire vertebral columns have been post-fixed for 4 hours in the same fixative solution and the spinal cords were later pulled out from it. Next, cervical, thoracic and lumbar tracts were dissected and fixed with different protocols. The protocols differ in the aldehydes, osmium tetroxide and reduced osmium tetroxide, uranyl acetate concentrations and times of treatment (see Supplementary Table 2). The optimized protocol is summarized in Table 1.

#### Large-size sample

Three Wistar rats, deeply anesthetized, were sacrificed by perfusion with an aldehydes fixative solution. The extraction of the spinal cord has been performed as described for the mouse samples. The chemical protocols tested, similarly to the medium-size samples, differ in the aldehydes, osmium tetroxide and reduced osmium tetroxide, uranyl acetate concentrations and times of treatment (Supplementary Table 3). The optimized protocol is summarized in Table 1.

All the fixed samples were further treated as previously described^12^. Briefly, samples were stained with uranyl acetate 0.5% or 3% in ethanol solution (20%), dehydrated in a growing series of ethanol and embedded in epoxy resin (EMbed 812 Kit, EMS), which was polymerized for 48 h at 60 °C.

### X-ray computed microtomography (micro-CT)

Embedded samples were analyzed with synchrotron X-ray phase contrast micro-CT. Experiments were performed at the SYRMEP beamline of the Elettra light source (Basovizza, Trieste)^23^ using Propagation Based Imaging (PBI) modality^24^.

All our samples were imaged using the white/pink imaging setup of the beamline at an average X-ray energy of 20 keV. Sciatic nerve and spinal cord samples were visually aligned with respect to the sample holder so that their longitudinal axes were as parallel as possible to the rotation axis. For small-size sample 1800 projections (each scan) were acquired over 180° rotation with a sample-to-detector distance of 8 cm. The resulting isotropic voxel size is 2.15 µm, and a single scan is sufficient because the sample is smaller than the field of view.

The spinal cord samples were scanned according to the so-called half acquisition (or extended field-of-view), where the rotation axis is close to one side of the detector field-of-view and a 360° rotation is considered. In this case, being the sample only slightly bigger than the detector field-of-view, 2400 projections (each scan) were considered with a sample-to-detector distance of 15 cm.

The isotropic voxel size is 2 µm, and multiple scans at different heights are required to image the whole sample. We acquired the second scan overlapping 500 microns with respect to the previous one. A phase retrieval pre-processing filter^19^ was applied, prior to the reconstruction, to decouple phase from absorption contribution. Tomographic reconstruction of the series of 2D projections was performed by using the SYRMEP Tomo Project software, developed at the SYRMEP beamline^25^. Filtered Back Projection (FBP) was used and common image processing was adopted to perform the required steps of flat fielding and image alignment with respect to the actual center of rotation^26^. Ring removal filters^27^ were also applied to compensate the inhomogeneity of pixel response caused by the detector used at binning 1 × 1. Further visual inspection of the reconstructed virtual stack was performed with the ImageJ software with the 3D Volume Viewer and the 3D Viewer plugins.

### Sectioning

After the micro-CT scan, the embedded samples were sectioned at room temperature in thick slices for light microscopy (LM) observation and thin ones for TEM analysis.

Sections for LM (500 nm thickness) were placed on glass slides, stained with 0.1% methylene blue and 0.1% toluidine blue in phosphate buffer 240 mM pH 7.4.

Sections for the TEM analysis (90 nm thickness) were placed on copper grids (G300Cu – EMS). The sectioning was performed using an ultramicrotome (UC7, Leica Microsystem, Vienna, Austria) equipped with a 45° diamond knife (DiATOME, Nidau, Switzerland), in which the samples were mounted so that their cross sections were perpendicular to the rotation axis of the micro-CT.

The exposition of the embedded samples to a strong X-ray beam during the tomographic process slightly darkened the resin but had no effects on the ultrastructure as confirmed by comparing sections cut before and after the tomographic process.

### Optical microscopy and TEM

Sections for optical microscopy were imaged with an optical microscope (DM750, Leica Microsystem, Vienna, Austria), equipped with an ICC50HD (Leica Microsystem, Vienna, Austria) digital camera. We used 10X HI PLAN (NA = 0.25) and 40X HI PLAN (NA = 0.65) objectives (Leica Microsystem, Vienna, Austria).

For the TEM analysis, sections were observed with a Zeiss LIBRA 120 Plus operating at 120 KeV equipped with an in-column omega filter, for the energy filtered imaging, and with a bottom mounted 6-bit 2k × 2k CCD camera (TRS) in bright field mode, energy filtered with a 20 eV slit on the zero loss peaks.

### Source and type of reagents/solutions

- 4% Paraformaldehyde: powder paraformaldehyde (Sigma-Aldrich powder, 95%) is dissolved in MilliQ H_2_O by heating at a temperature lower than 60 °C and stirring until the solution is completely clear. Thereafter the pH value has been regulated (7.2 (±0.2)).
- 8% Paraformaldehyde: powder paraformaldehyde (Sigma-Aldrich powder, 95%) is dissolved in PBS buffer by heating at a temperature lower than 60 °C and stirring until the solution is completely cleared. Thereafter the pH value has been regulated (7.2 (±0.2)).
- 25% Glutaraldehyde aqueous solution (Electron Microscopy Sciences (EMS)).
- 37% Formaldehyde: stock solution in PBS is prepared starting from the powder Paraformaldehyde (Sigma-Aldrich powder, 95%) at a temperature of 60 °C adding NaOH 1 M (1–2% V/V). After the solubilization, pH value is regulated with HCl to the value of 7.4 (±0.1).
- 4% Osmium tetroxide aqueous solution (EMS).
- 4% Potassium ferricyanide: the solution 4% potassium ferricyanide is prepared from a powder (ACS reagent, ≥99%, powder, <10 μm (Sigma-Aldrich)) then diluted in H_2_O milliQ; the solution has been stocked in dark at +4 °C.
- 3% Uranyl acetate: the solution 3% uranyl acetate (Uranyl Acetate, Reagent, A.C.S. - EMS) is prepared from a powder diluted in 20% ethanol aqueous solution; the solution has been stocked in dark at +4 °C.
- Propylene oxide (EM Grade (EMS)).
- Epoxy resin EMbed 812 Kit (EMS). The resin has been prepared in two stock solution: the first one is made of 12.5 gr DDSA + 11.75 gr EPON 812; the second solution is made with 12.05 gr NMA + 18.5 gr EPON 812. Each solution has been well agitated and they were stocked at 4 °C; before the use, the two resins were mixed in equal parts, and it was added 2% accelerator; the final solution has been mixed and the bubbles were removed with a sonicator.

## Electronic supplementary material

Supplementary Information 
