# Supplementary material for: Size and specimen-dependent strategy for x-ray micro-ct and tem correlative analysis of nervous system samples
Source: Sci Rep. 2017 Jun 6;7:2858. doi: 10.1038/s41598-017-02998-1 (PMC5460131; doi:10.1038/s41598-017-02998-1)
Supplement: Supplementary file 1 — Supplementary Information [file 41598_2017_2998_MOESM1_ESM.doc]

**SIZE AND SPECIMEN-DEPENDENT STRATEGY FOR X-RAY MICRO-CT AND TEM CORRELATIVE ANALYSIS OF NERVOUS SYSTEM SAMPLES**

P. Parlanti^1,2,$^, V. Cappello^2,$,*^, F. Brun^3,4,5^, G. Tromba^5^, R. Rigolio^6^, I. Tonazzini^7,8^, M. Cecchini^7^, V. Piazza^2^, M. Gemmi^2^

**SUPPLEMENTARY INFORMATION**


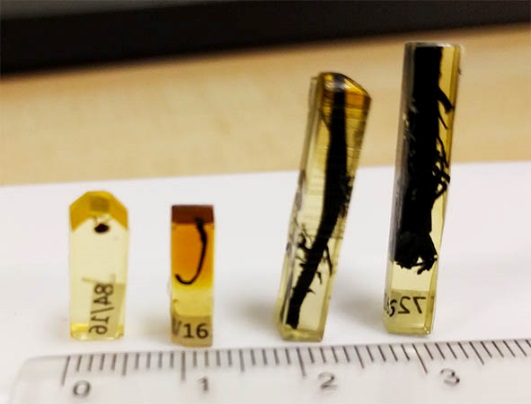
**Supplementary Figure 1.** Representative picture of the samples named as: 1) ideal-size; 2) small-size; 3) medium-size; 4) large-size (respectively from the left to the right).


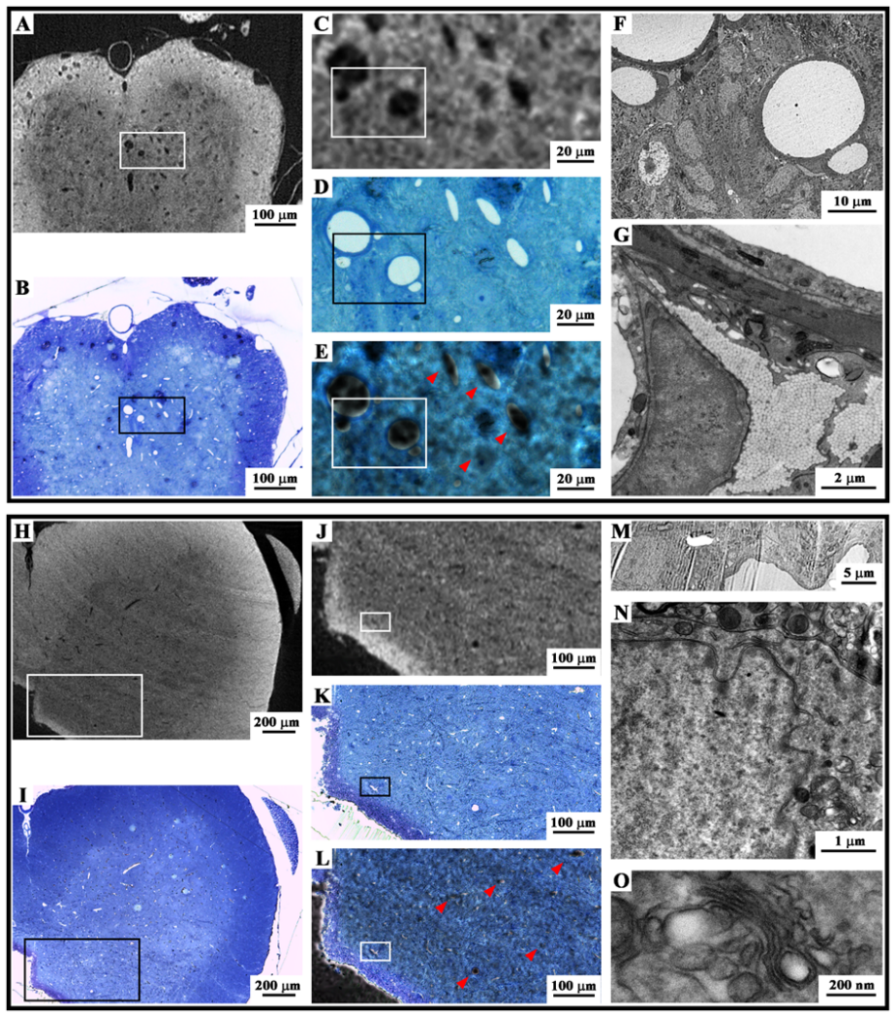
**Supplementary Figure 2. Correlation procedure in larger samples.** Upper panel: (A,B) representative virtual and real slices of mice spinal cord. (C,D) Higher magnification of the boxed region. (E) Merged image of C and D indicates the correct alignment; arrowheads indicate the correlation points used for the alignments of virtual and optical sections. (F,G) Respectively, an electron micrograph at low and higher magnification, of the boxed region in C-E. Lower panel: (H,I) Representative virtual and real slices of rat spinal cord (J, K). Higher magnification of the boxed region (L). Merged image of J and K indicates the correct alignment; arrowheads indicate the correlation points used for the alignments of virtual and optical sections. (M,O) Respectively, an electron micrograph at low and higher magnifications, of the boxed region in J-L. TEM analysis clearly confirms the ultrastructural preservation of the sample.


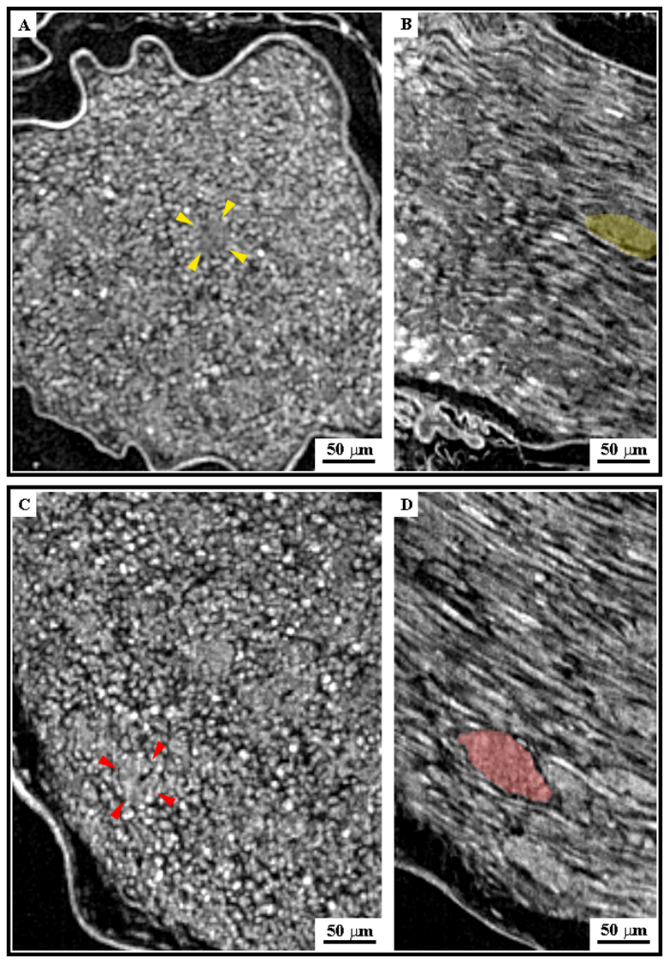
**Supplementary Figure 3. Globoid cells identification.** (A,C) Virtual slices of a sciatic nerve treated with the small-size protocol, in which it is possible to identify infiltrating cells; two representative cells are indicated, respectively, with yellow and red arrowheads. (B,D) Longitudinal views of the same sample in which the cells located in A and C are visible and colored, in yellow and red respectively.

| **SMALL SIZE SAMPLE - MICE SCIATIC NERVES** | | | | | |
| --- | --- | --- | --- | --- | --- |
|  | **Protocol I ideal size** | **Protocol II** | **Protocol III** | **Protocol IV** | **Protocol V optimized small size** |
| **Protein Fixation (perfusion)** | Para 4% + Gluta 0.1% in PBS | Para 4% + Gluta 0.1% in PBS | Para 4% + Gluta 0.1% in PBS | Para 4% + Gluta 2.5% in PBS | Para 4% + Gluta 0.1% in PBS |
| **Protein Fixation** | Overnight Gluta 2% in NaCaco Buffer | Overnight Gluta 2% in NaCaco Buffer | Overnight Gluta 2% + Para 2% in NaCaco Buffer | Overnight Gluta 2% + Para 1% + Form 1% in NaCaco Buffer | Overnight Gluta 2% + Para 1% + Form 1% in NaCaco Buffer |
| **Lipid Fixation** | 1h OsO_4_ 1% + K_3_Fe(CN)_6_ 1% in NaCaco Buffer | 1h OsO_4_ 2% in NaCaco Buffer | 1h OsO_4_ 1% + K_3_Fe(CN)_6_ 1% in NaCaco Buffer | 1h OsO_4_ 1% + K_3_Fe(CN)_6_ 1% in NaCaco Buffer | 1h OsO_4_ 1% + K_3_Fe(CN)_6_ 1% in NaCaco Buffer |
| **Contrast** | 1h UA 3% in ethanol 20% | 1h UA 3% in ethanol 20% | 1h UA 3% in ethanol 20% | 1h UA 3% in ethanol 20% | 1h UA 3% in ethanol 20% |

**Supplementary Table 1.** The table reports all the embedding procedures tested on the mice sciatic nerves, starting from the ideal-size protocol (Protocol I). The optimized protocol is described in the last column of the table (Protocol V).

| **MEDIUM SIZE SAMPLE - MICE SPINAL CORDS** | | | | | | | | | | | |
| --- | --- | --- | --- | --- | --- | --- | --- | --- | --- | --- | --- |
|  | **Protocol I optimized small size** | **Protocol II** | **Protocol III** | **Protocol IV** | **Protocol V** | **Protocol VI** | **Protocol VII** | **Protocol VIII** | **Protocol IX** | **Protocol X** | **Protocol XI optimized medium size** |
| **Protein Fixation (perfusion)** | Para 4% +  Gluta 0.1% in PBS | Para 4% + Gluta 0.1% in PBS | Para 4% + Gluta 0.1% in PBS | Para 4% + Gluta 0.1% in PBS | Para 4% + Gluta 2.5% in PBS | Para 4% + Gluta 2.5% in PBS | Para 4% + Gluta 2.5% in PBS | Para 4% + Gluta 2.5% in PBS | Para 4% + Gluta 0.1% in PBS | Para 4% + Gluta 0.1% in PBS | Para 4% + Gluta 0.1% in PBS |
| **Protein Fixation** | Overnight  Gluta 2% + Para 1% +  Form 1% in NaCaco Buffer | Overnight  Gluta 2% + Para 2% in NaCaco Buffer | Overnight  Gluta 2% in NaCaco Buffer | Overnight  Gluta 2% in NaCaco Buffer | Overnight  Gluta 2% + Para 1% +  Form 1% in NaCaco Buffer | Overnight  Gluta 2% + Para 2% in NaCaco Buffer | Overnight  Gluta 2% in NaCaco Buffer | Overnight  Gluta 2% in NaCaco Buffer | Overnight  Gluta 2.5% + Para 1% +  Form 1% in NaCaco Buffer | Overnight  Gluta 2.5% + Para 1% +  Form 1% in NaCaco Buffer | Overnight  Gluta 2.5% + Para 1% + Form 1% in NaCaco Buffer |
| **Lipid Fixation** | 1h OsO_4_ 1% + K_3_Fe(CN)_6_ 1% in NaCaco Buffer | 1h OsO_4_ 1% + K_3_Fe(CN)_6_ 1% in NaCaco Buffer | 1h OsO_4_ 1% + K_3_Fe(CN)_6_ 1% in NaCaco Buffer | 1h OsO_4_ 2% in NaCaco Buffer | 1h OsO_4_ 1% + K_3_Fe(CN)_6_ 1% in NaCaco Buffer | 1h OsO_4_ 1% + K_3_Fe(CN)_6_ 1% in NaCaco Buffer | 1h OsO_4_ 1% + K_3_Fe(CN)_6_ 1% in NaCaco Buffer | 1h OsO_4_ 2% in NaCaco Buffer | 8h OsO_4_ 1% + K_3_Fe(CN)_6_ 1% in NaCaco Buffer | 8h OsO_4_ 1% + K_3_Fe(CN)_6_ 1% in NaCaco Buffer | 8h OsO_4_ 1% + K_3_Fe(CN)_6_ 1% in NaCaco Buffer |
|  |  |  |  |  |  |  |  |  | Overnight NaCaco Buffer |  | Overnight  OsO_4_ 1% in NaCaco Buffer |
| **Contrast** | 1h UA 3%  in ethanol 20% | 1h UA 3%  in ethanol 20% | 1h UA 3%  in ethanol 20% | 1h UA 3%  in ethanol 20% | 1h UA 3%  in ethanol 20% | 1h UA 3%  in ethanol 20% | 1h UA 3%  in ethanol 20% | 1h UA 3%  in ethanol 20% | 1h UA 3%  in ethanol 20% | Overnight  UA 0.5% in ethanol 20% | 1h UA 3%  in ethanol 20% |

**Supplementary Table 2.** The table reports all the embedding procedures tested on the mice spinal cords, starting from the small-size protocol (Protocol I). The optimized protocol is described in the last column of the table (Protocol XI).

| **LARGE SIZE SAMPLE - RATS SPINAL CORDS** | | | | | | | | | | | |
| --- | --- | --- | --- | --- | --- | --- | --- | --- | --- | --- | --- |
|  | **Protocol I optimized medium size** | **Protocol II** | **Protocol III** | **Protocol IV** | **Protocol V** | **Protocol VI** | **Protocol VII** | **Protocol VIII** | **Protocol IX** | **Protocol X** | **Protocol XI optimized large size** |
| **Protein Fixation (perfusion)** | Para 4% +  Gluta 0.5% in PBS | Para 4% + Gluta 0.5% in PBS | Para 4% + Gluta 0.5% in PBS | Para 4% + Gluta 0.5% in PBS | Para 4% + Gluta 0.5% in PBS | Para 4% + Gluta 0.5% in PBS | Para 4% + Gluta 0.5% in PBS | Para 4% + Gluta 0.5% in PBS | Para 4% + Gluta 0.5% in PBS | Para 4% + Gluta 0.5% in PBS | Para 4% + Gluta 0.5% in PBS |
| **Protein Fixation** | Overnight  Gluta 2.5% + Para 1% +  Form 1% in NaCaco Buffer | Overnight  Gluta 2.5% + Para 1% +  Form 1% in NaCaco Buffer | Overnight  Gluta 2.5% + Para 1% +  Form 1% in NaCaco Buffer | Overnight  Gluta 2.5% + Para 1% +  Form 1% in NaCaco Buffer | Overnight  Gluta 2.5% + Para 1% +  Form 1% in NaCaco Buffer | Overnight  Gluta 2.5% + Para 1% +  Form 1% in NaCaco Buffer | Overnight  Gluta 2.5% + Para 1% +  Form 1% in NaCaco Buffer | Overnight  Gluta 2.5% + Para 1% +  Form 1% in NaCaco Buffer | Overnight  Gluta 2.5% + Para 1% +  Form 1% in NaCaco Buffer | Overnight  Gluta 2.5% + Para 1% +  Form 1% in NaCaco Buffer | Overnight  Gluta 2.5% + Para 1% + Form 1% in NaCaco Buffer |
| **Lipid Fixation** | 8h OsO_4_ 1% + K_3_Fe(CN)_6_ 1% in NaCaco Buffer | 8h OsO_4_ 1% + K_3_Fe(CN)_6_ 1% in NaCaco Buffer | 8h OsO_4_ 1% + K_3_Fe(CN)_6_ 1% in NaCaco Buffer | 8h OsO_4_ 1% + K_3_Fe(CN)_6_ 1% in NaCaco Buffer | 8h OsO_4_ 2% in NaCaco Buffer | 8h OsO_4_ 2% + K_3_Fe(CN)_6_ 1% in NaCaco Buffer | 8h OsO_4_ 1% + K_3_Fe(CN)_6_ 1% in NaCaco Buffer | 8h OsO_4_ 1% + K_3_Fe(CN)_6_ 1% in NaCaco Buffer | 8h OsO_4_ 1% + K_3_Fe(CN)_6_ 1% in NaCaco Buffer | 8h OsO_4_ 1% + K_3_Fe(CN)_6_ 1% in NaCaco Buffer | 8h OsO_4_ 2% + K_3_Fe(CN)_6_ 1% in NaCaco Buffer |
|  | Overnight  OsO_4_ 1% in NaCaco Buffer | Overnight NaCaco Buffer |  | Overnight NaCaco Buffer | Overnight NaCaco Buffer | Overnight NaCaco Buffer | Overnight  OsO_4_ 2% in NaCaco Buffer | Overnight  OsO_4_ 2% + K_3_Fe(CN)_6_ 1% in NaCaco Buffer | 1 day and overnight OsO_4_ 1% in NaCaco Buffer | 1 day  OsO_4_ 2% in NaCaco Buffer | Overnight  OsO_4_ 1% + K_3_Fe(CN)_6_ 1% in NaCaco Buffer |
|  |  |  |  | 1h OsO_4_ 1% in NaCaco Buffer |  |  |  |  |  | Overnight NaCaco Buffer |  |
| **Contrast** | 1h UA 3%  in ethanol 20% | 1h UA 3%  in ethanol 20% | Overnight  UA 0.5% in ethanol 20% | 1h UA 3%  in ethanol 20% | 1h UA 3%  in ethanol 20% | 1h UA 3%  in ethanol 20% | 1h UA 3%  in ethanol 20% | 1h UA 3%  in ethanol 20% | 1h UA 3%  in ethanol 20% | 1h UA 3%  in ethanol 20% | 1h UA 3%  in ethanol 20% |

**Supplementary Table 3.** The table reports all the embedding procedures tested on the rats spinal cords, starting from the medium-size protocol (Protocol I). The optimized protocol is described in the last column of the table (Protocol XI).
